# *Happiness* vs. *Wellness* During the Recovery Process in High Performance Sport

**DOI:** 10.3389/fphys.2018.01598

**Published:** 2018-12-19

**Authors:** Julio Calleja-González, Nicolas Terrados, Rafael Martín-Acero, Carlos Lago-Peñas, Igor Jukic, Juan Mielgo-Ayuso, Diego Marqués-Jiménez, Anne Delextrat, Sergej Ostojic

**Affiliations:** ^1^Department of Physical Education and Sports, University of the Basque Country (UPV-EHU), Bilbao, Spain; ^2^Department of Functional Biology, University of Oviedo, Oviedo, Spain; ^3^University of Vigo, Vigo, Spain; ^4^School of Kinesiology, University of Zagreb, Zagreb, Croatia; ^5^Department of Biochemistry, Molecular Biology and Physiology, Universidad de Valladolid, Soria, Spain; ^6^Oxford Brookes University, Oxford, United Kingdom; ^7^University of Novi Sad, Novi Sad, Serbia

**Keywords:** happiness, wellness, recovery, performance, elite, sport

In the last June 2017, Dr. Martin Buchheit alerted the Sport's community with an editorial entitled “Houston, we still have a problem,” (Buchheit, 2017) published in one of the most impact scientific journal the field of sports sciences. In that document, he explained that some of the most used methods in elite sports with a high level of evidence (1a) are not linking of athletes. However, other methods, without any evidence yet, are usually used among them. Although these used strategies present a high placebo effect among elite athletes, the implementation is a controversial topic in sports competition. Besides the physician staff have the control about its use, it is their responsibility to show leadership to create a satisfying and productive working environment for the future colleagues (Kimura, 2016), but these practices have not been studied yet.

In particular, some of these techniques and products used in order to enhance the recovery process are described in the scientific literature. For example, regarding ergogenic aids, only 5 supplements with 1a level of evidence improve performance according to the Australian Institute of Sport (caffeine, betalanine, bicarbonate, creatine, and nitrate (http://www.ausport.gov.au/ais/nutrition/supplements), although professional teams spend a lot of money using products without evidences (Bishop, 2010). With relation to the dip in frozen water, we have gained time and comfort. Currently athletes dip during 10 min at 11–15°C, compared with old technologies in with dips at 0°C during longer periods of time (Anderson et al., 2017). On the other hand, massage is also a technique widely used in the world of sports and that sportsmen like, but meta-analysis studies did not show effect on sports recovery (Poppendieck et al., 2016). Recently, trips are one of the hot topics in sports science. Every time the athletes compete more frequently and must make more trips during periods of high competitive density. A priori being able to guarantee an adequate recovery with hygiene of the circadian rhythm is of vital importance throughout a season. Therefore, the fact of sleeping the day before and after the game at the destination should be a common practice in order to ensure recovery, since the reduced amount and quality of sleep are mainly evident after a game in elite players (Fullagar et al., 2016). However, athletes prefer to break their biological rhythm, arrive at dawn to the place of origin, in order to sleep at home with their family. In that way objective data showed trends toward longer sleep length at home (Baulk and Fletcher, 2012). In this case accelerating the come back home despite not being the most convenient is proposed in the part of the season where there are no important periods and as we are getting closer to competitions of interest having to spend the night in the hotel.

In all these situations the same reflection occurs when implementing or not the different recovery methods, in relation to the level of evidence in order to obtain a wellness, or on the contrary, if the athlete really prefers, with what we would obtain an evident happiness.

Athlete self-report measures include perceptions of wellbeing (e.g., fatigue) and psychological variables (e.g., mood) which are influenced by both training and non-training stressors (Kellmann, 2010).

According to Corbin (2009) “emotional wellness is a person's ability to cope with daily circumstances and to deal with personal feelings in a positive, optimistic, and constructive manner”.

Erickson refers to the so-called Wellness Wheel; a model which “portrays a balance between six dimensions of life and health—physical, social, environmental, emotional, spiritual, and intellectual.” Consequently, it is vital, for sportsmen, to keep all the wellness dimensions in balance in order to produce better performance (Erickson, 2012).

In contrast, Happiness is of great importance to most people and has been found to be a highly valued goal in most societies (Fisher, 2010).

Happiness underlying factors are considerable from two dimensions: endogenic factors (biological, cognitive, personality, and ethical sub-factors) and exogenic factors (behavioral, socio-cultural, economical, geographical, life events, and aesthetics sub-factors). Among all endogenic factors, biological sub-factors are the significant predictors of happiness (Dfarhud et al., 2014). Besides, improving staff happiness may contribute to increase in moral and counter burnout (Baruch et al., 2013). In that way, investigation proposes that for many people happiness is being able to make the practices of everyday life work, such that positive feelings control over negative feelings follow-on from daily hassles (Olsson et al., 2013).

Although one of the most important elements of performance and exercise is rest (Counting on the placebo effect as an interesting phenomenon to consider) and a significant predictor of successful performance (Uphill et al., 2014), confirming the previous statement of Totterdell (Totterdell, 1999); players perform better when they are in positive moods. For that reason, the balance between “happiness and wellness” in high sports performance, is presented as an exciting challenge in the coming years, where the cost-benefit should be the main measurement parameter, considering that happiness is strongly correlated with perceived good health (Sabatini, 2014), although to date for the best of our knowledge, there is no scientific evidence about it. Probably, in the initial phases of the season, we could give greater importance to happiness, because the happiness was the attainment of a worthy life (Stearns, 2012), and as we approach the important competitions generate a culture of wellness.

Time will tell, but we must remember that once again, we think this applies to all jobs within social services as we are working with people and impacting on their lives. Therefore, we could say: “Houston we still have a problem” but we could add., “Remember that “We are physicians, but first and foremost, human beings”” (Kimura, 2016).

## Author Contributions

JC-G and NT: First Idea; RM-A: Review; CL-P: Coauthor; IJ: Review; JM-A: Writer; DM-J: References; AD: Language; SO: Main director.

### Conflict of Interest Statement

The authors declare that the research was conducted in the absence of any commercial or financial relationships that could be construed as a potential conflict of interest.
